# Partner gaze shapes the relationship between symptoms of psychopathology and interpersonal coordination

**DOI:** 10.1038/s41598-024-65139-5

**Published:** 2024-06-21

**Authors:** M. C. Macpherson, A. J. Brown, R. W. Kallen, M. J. Richardson, L. K. Miles

**Affiliations:** 1 School of Psychological Science, University of Western Australia, Perth, Australia; 2 School of Psychological Sciences and Performance and Expertise Research Centre, Macquarie University, Sydney, Australia

**Keywords:** Human behaviour, Psychology

## Abstract

Interpersonal coordination is a key determinant of successful social interaction but can be disrupted when people experience symptoms related to social anxiety or autism. Effective coordination rests on individuals directing their attention towards interaction partners. Yet little is known about the impact of the attentional behaviours of the partner themselves. As the gaze of others has heightened salience for those experiencing social anxiety or autism, addressing this gap can provide insight into how symptoms of these disorders impact coordination. Using a novel virtual reality task, we investigated whether partner gaze (i.e., direct vs. averted) influenced the emergence of interpersonal coordination. Results revealed: (i) spontaneous coordination was diminished in the averted (cf. direct) gaze condition; (ii) spontaneous coordination was positively related to symptoms of social anxiety, but only when partner gaze was averted. This latter finding contrasts the extant literature and points to the importance of social context in shaping the relationship between symptoms of psychopathology and interpersonal coordination.

## Introduction

Interpersonal coordination is crucial for fostering social connections. Aligning actions with others promotes affiliative behaviour^1^, but is undermined by negative contextual factors that harm social interaction (e.g., rudeness, argument, poor mental health^2–4^). Effective coordination relies on information exchange between individuals, typically via visual attention^5,6^. Research documenting this effect however has concentrated on the attentional patterns of individual participants^6^, with less focus given to the concurrent behaviour of their interaction partner. This prompts an important question: does the gaze of an interaction partner impact the emergence of interpersonal coordination?

Related research indicates that psychopathologies known to impact reciprocal social behaviour, in particular social anxiety disorder (SAD) and autism spectrum disorder (ASD), reduce interpersonal coordination^4,7^. Importantly, these disorders are also characterised by atypical responses to gaze cues that can undermine social exchange^8,9^. With these findings in mind, the current research employs virtual reality (VR) to examine: (i) how the gaze patterns of an interaction partner influence the emergence of interpersonal coordination; and (ii) whether partner gaze impacts the relationship between subclinical variation in symptoms of SAD and/or ASD, and interpersonal coordination.

### Attention, social context, and interpersonal coordination

How people attend to others plays a pivotal role in the emergence of interpersonal coordination. Contemporary models of motor control (e.g., Haken-Kelso-Bunz Equation^10^) show that, along with similar movement frequencies, the degree to which components of a system are coupled governs both spontaneous and intentional instances of coordination^11^. Coupling dictates the extent to which information exchange occurs between system components. Within interpersonal contexts coupling is typically perceptual—directing attention to an interaction partner provides the information required for coordination to emerge. Varying the strength of this attentional coupling leads to concomitant variation in coordination. For instance, coordination is enhanced when movements are dynamically tracked (cf. attention fixed on a stationary point) and central to one’s attentional focus^6,12^. To date this work has been concerned with manipulating the visual attentional patterns of individuals. Little consideration has been given to how the gaze behaviour of an interaction partner may also impact the emergence of coordination.

The attentional patterns of others can have profound effects on the social context in which an interaction unfolds^13,14^. At the most fundamental level, a partner’s gaze direction can specify the focus of their attention (e.g., self vs. other) and affiliative goals (e.g., approach vs. avoid^15^). For instance, direct gaze is typically associated with positive interactions, promoting approach behaviour, and enhancing perceptions of trustworthiness^16,17^. In contrast, averted gaze is associated with avoidance, disinterest, and exclusion^16,18^, and can lead to aggressive responses^19^. Simply being looked at, or not, can polarise social context, shifting behavioural tendencies from prosocial and affiliative, to pro-self and exclusionary.

It follows therefore that an interaction partner’s gaze behaviour may, via shaping social context, impact the emergence of interpersonal coordination. When negative social contexts arise through aversive or ill-mannered behaviour (e.g., being rude, late, or argumentative), lower levels of coordination are observed^2,3^. On the other hand, in more affiliative contexts (e.g., speed dating), a higher level of behavioural coordination is a robust predictor of positive interpersonal goals (e.g., romantic interest^20^). Social context, in particular the evaluative and affective elements, can direct the emergence of interpersonal coordination.

On this basis, it is tempting to adopt a straightforward hypothesis: the exclusion and disinterest implied by the averted gaze of an interaction partner will result in reduced levels of coordination. Yet, being excluded from an interaction can trigger diverse responses, including withdrawal and aggression, or alternatively, *increases* in prosocial and ingratiating behaviour^21^. As such, people could be expected to coordinate *more* with an interaction partner who is not paying them attention, in service of important social motives (i.e., the need to belong^22^). Indeed, analogous effects have been reported in the social coordination literature. For instance, participants who interacted with an out-group member showed higher levels of interpersonal coordination compared to those who had an equivalent in-group interaction^23^. Importantly, this increase was only observed when a subsequent meeting with that out-group member was anticipated, indicative of an attempt to generate rapport and reduce awkwardness prior to the next interaction. In this sense, averted gaze may lead to greater levels of coordination as a means to reduce social distance and ingratiate others.

### Gaze, symptoms of psychopathology, and interpersonal coordination

To gain insight into the relationship between partner gaze and interpersonal coordination, it is also important to consider disorders related to impairments in reciprocal social behaviour. A growing body of literature demonstrates a robust link between psychopathology and coordination^4,7^. For individuals diagnosed with disorders of social interaction (e.g., SAD, ASD), interpersonal coordination is disrupted. Importantly, recent work indicates this pattern of effects extends beyond diagnostic categories—subclinical symptoms of SAD or ASD also negatively impact interpersonal coordination^24,25^. In line with these findings and contemporary continuum-based models of psychopathology^26^, the present research employs a subclinical sample.

Of relevance to the current enquiry, symptoms of ASD and SAD are associated with atypical responses to the gaze of others. In ASD, individuals typically display reductions in mutual gaze, an avoidance behaviour thought to arise from differences in the salience of eye contact^9^. In SAD, individuals show a complex pattern of gaze vigilance and avoidance, initially orientating towards the eyes of an interaction partner (vigilance), before rapidly averting their own gaze so as to deter eye contact (avoidance) with that person^17^. In this respect, the direct gaze of an interaction partner may exacerbate the relationship between symptoms of these disorders and interpersonal coordination, leading to lower levels of coordination among individuals with heightened levels of SAD/ASD.

### Assessing partner influences on interpersonal coordination

Capturing the influence of an interaction partner’s behaviour on coordination poses methodological challenges. Previous research has sacrificed experimental control by pairing participants with a confederate^2^ or experimenter^27^. However, posing as a participant introduces unwanted variability that may bias behaviour and preclude replication. Immunity to the implicit influence of social norms is beyond even the most well-trained confederate^1^, as is the precise repetition of behaviour^28^. As an alternative, coordination researchers have also focused on pairs of naïve participants, an approach that presents a separate issue. Factors that are typically considered at the individual-level of analysis (e.g., mental health symptoms), must be combined to enable consideration at a collective-level (i.e., at the level of coordination). To do so, researchers have employed averaging^24^, differencing^29^, or multiplication^30^ of individual symptom levels. These approaches are, however, blunt and unlikely to accurately reflect the complex non-linear interactions inherent to dynamic social exchange.

To address these problems, researchers have turned to using virtual reality (VR) in interpersonal coordination research^31^. VR offers high-fidelity immersive environments that allow precise control over experimental manipulations and unobtrusive means to capture behaviour. Using pre-programmed virtual avatars in place of confederates eliminates unwanted behavioural variability and other extraneous factors, thereby ensuring precise procedural replication is possible. Further, because no actual dyad is required, the analytical issues associated with estimating individual characteristics (e.g., mental health symptoms) at the collective level are avoided. Although concerns have been raised regarding the validity of VR as a research tool^32^, both the dynamical properties^33^ and the characteristic social outcomes^34^ of interpersonal coordination are consistent between virtual and face-to-face encounters. Indeed, VR offers a promising avenue to explore interpersonal coordination in a realistic yet well-controlled research context.

### Current research

The gaze behaviours of an interaction partner convey an abundance of social information, yet little is known about their influence on the emergence of interpersonal coordination. To address this issue, in VR participants performed a simple movement task with an avatar that was allegedly controlled by another participant, but in reality, displayed pre-recorded movements. The avatar either looked directly at the participant (direct gaze condition) or looked away (averted gaze condition). Throughout each virtual interaction, we captured participants’ arm movements to estimate coordination, and their gaze patterns as an index of their attention. Quantifying participant attentional behaviour provided an important complement to the focus of the present work (i.e., partner gaze patterns), by enabling consideration of a factor known to govern coordination^6,12^. Participants also completed measures of subclinical variation in SAD and ASD.

To extend the scope of the current research, we manipulated and measured core dynamical properties of interpersonal coordination. Specifically, all participants completed uninstructed (i.e., spontaneous) and instructed (i.e., intentional) coordination trials as a means to vary task dynamics^6^. Although an influence of symptoms of SAD and ASD has been documented for both spontaneous^29^ and intentional^4,35^ coordination, recent empirical findings point to coordination stability as a potential boundary condition of this relationship^25^. Given spontaneous coordination is dynamically less stable^6^, manipulating task dynamics provides means to further evaluate the factors that promote and/or inhibit the relationship between SAD/ASD and coordination.

### Predictions

The extant literature indicates two likely outcomes when people feel excluded by others – they either withdraw from the interaction^2,3^ or escalate their affiliative behaviour^21^. On this basis, the social disinterest implied by averted partner gaze may reduce *or* enhance interpersonal coordination. We also anticipate a negative relationship between SAD/ASD and coordination^4,7^. Given the atypical responses to eye contact associated with SAD/ASD^9,17^, we expect that this relationship will be stronger in the direct gaze condition. We also expect the influence of partner gaze and SAD/ASD to be stronger during spontaneous (cf. intentional) coordination given the inherent differences in dynamic stability^5^.

In terms of participant attentional patterns, given that coordination relies on a perceptual coupling^11^, we expect that time spent looking at the interaction partner will boost spontaneous and intentional coordination. Taking a more exploratory stance, due to characteristic patterns of gaze avoidance^9,17^, we also expect a negative relationship between symptoms of SAD/ASD and time spent looking at the partner. Finally, because of reduced time spent looking at the partner, we expect that direct partner gaze and higher levels of SAD/ASD will reduce coordination in both spontaneous and intentional conditions.

## Method

### Participants and design

The target sample size was determined a priori using G*Power (v 3.1.9.7^36^). Drawing from previous research that manipulated social factors and assessed the impact on interpersonal coordination^3,23,37^ we conservatively anticipated an effect size of *f* = 0.25. To provide 80% power to detect a significant mixed-effect interaction (α = 0.05) required a minimum sample size of 122 participants (61 per condition). This sample size also provides sufficient statistical power to detect correlations between questionnaire measures and coordination greater than *r* = 0.2. Comparable work has^24^ reported equivalent correlations that comfortably exceeded this value (LSAS: *r* = 0.38; AQ: *r* = 0.35)^34^. To maintain a conservative stance and allow for missing or incomplete data, we set a stopping rule of 150 participants in total. Ultimately, 147 undergraduates took part in return for course credit, at which point the available participant pool was exhausted. Only individuals aged 18 years or over with no injury or impairment that impacted arm movement were eligible. Eleven participants were excluded after indicating they did not believe the cover story (i.e., that the avatar was controlled by another participant) and technical problems meant no movement data was available for three participants. Within the final sample (*n* = 133; 94 female, 39 male; aged 18–50 years, *M* = 20.1 years, *SD* = 4.5 years), one participant was also missing movement data, but only for the intentional coordination trial. Unsuccessful eye-tracking calibration resulted in missing gaze data for one participant for the spontaneous coordination condition, and six participants for both coordination conditions. All remaining data from these participants were retained.

The experiment employed a 2 (avatar gaze: direct vs. averted) × 2 (coordination: spontaneous vs. intentional) mixed design (with repeated measures on the second factor). Participants were randomly assigned to interact with an avatar who either looked at them (direct gaze condition, *n* = 68) or away from them (averted gaze condition, *n* = 65) for the duration of the interaction. To ensure that the instruction to coordinate in the intentional condition did not influence performance during the spontaneous condition, the order of the coordination conditions was fixed (i.e., spontaneous first). The research was approved by the Human Research Ethics Committee at the University of Western Australia and conducted in accordance with the Australian National Statement on Ethical Conduct in Human Research 2023. Informed consent was obtained from all participants prior to taking part.

### Procedure and materials

Participants were recruited to a study examining how people attend to themselves and others in virtual reality (VR). The study was described as involving a movement task with another participant, first in a virtual context, and subsequently in-person. Upon arrival participants were told that the other participant was present and located in a nearby laboratory. In reality the ‘other participant’ was a pre-programmed virtual avatar, and no face-to-face interaction took place. This cover story was modelled on previous work^23,37^ and intended to ensure naturalistic behaviour by giving participants the impression they were engaging in a real-time social exchange, (i.e., the avatar was being controlled by another person rather than being pre-programmed^23,41^). Additionally, the potential for a future in-person encounter also leads to more naturalistic social behaviour during virtual interactions (e.g., stronger adherence to social norms when attending to others^38^), hence the initial (false) indication of a face-to-face stage of the procedure subsequent to the virtual interaction.

Participants provided their basic demographic information (age and gender using a free-response format) before completing the Liebowitz Social Anxiety Scale (LSAS^39^), to assess symptoms of SAD (sample range = 9–119, *M* = 55.43, *SD* = 23.52), and the Autism Spectrum Quotient-Short (AQ^40^) to assess symptoms of ASD (sample range = 1–25, *M* = 9.99, *SD* = 4.83). The LSAS and the AQ have strong psychometric properties when used in community samples^40,41^, and have been routinely employed in interpersonal coordination research^4,24,25^. Where participants neglected to respond to an item, we replaced the missing value with their mean from the relevant subscale. This resulted in the replacement of 10 responses across the full sample (i.e., < 0.001%). As part of additional projects, participants also completed the Hospital Anxiety and Depression Scale (HADS^42^) and the State-Trait Anxiety Inventory (STAI^43^). At the completion of the procedure, participants completed the Embodiment Questionnaire^44^. We do not report these data here. To help maintain the cover story, while participants were completing the questionnaires, the experimenter briefly left the room to allegedly *“*check on the other participant*”*.

Next, participants were introduced to the VR system (Vive Pro Eye, HTC Corporation, Taiwan). They were fitted with a head-mounted display (HMD) to view the virtual environment, which was developed using the Unity3D Game Engine (v 2018.4.8f1). The HMD had dual OLED 3.5″ screens (1440 × 1600 pixels per eye, 110° × 106° field of view), and was equipped with an onboard eye-tracker (Tobii Pro Inc., Sweden, 120 Hz, accuracy 0.5° ~ 1.1°). Eye-tracking allowed estimates of participant attentional behaviour via defining two areas of interest pertaining to the avatar versus the rest of the room. Participants were given handheld controllers (Vive Pro 2018) that tracked movement with six degrees of freedom. When in the virtual environment, the controllers were represented as hands. Once familiar with the equipment, participants were reminded that they would be interacting with the other participant in VR, which would involve a simple arm movement task.

The VR procedure began with a 5-point eye-tracking calibration using the Super Reality (SR) runtime, followed by a practice trial intended to familiarise the participant with VR and to establish a target movement frequency. The practice trial took place in a generic grey virtual environment with no visual distractors. Participants were asked to perform arm curls (i.e., flexion/extension about the elbow) in time with a metronome (84 bpm) for 20 s. The metronome was played through the HMD headphones and participants held the controllers throughout the trial. The experimenter verbally corrected the participant if they did not perform the movement correctly (e.g., if they did not keep in time with the metronome or limited the range of their arm movements). All task instructions were presented visually in the HMD and reiterated verbally by the experimenter.

Next, participants were told that they would be performing two further arm curl trials, but this time while watching the other participant do the same. It was explained that both themselves and the other participant would be represented as virtual avatars that precisely reflected their respective real-world behaviour including arm, body, and head movements. They were placed into a virtual laboratory setting (5.34 m × 4.34 m) and told to perform the arm curls at the same pace as in the practice trial. It was stated that they should begin as soon as the other participant appeared, and to focus on forming an impression of them. As this served as the spontaneous coordination condition, no further instructions were given, and no mention was made of coordination. The trial lasted 90 s during which the avatar faced the participant (standing approximately 1.5 m away) and performed arm curls at the same pace as the practice trials. At the end of the 90 s the avatar disappeared, signalling the end of the trial. Following a short delay, the intentional coordination trial was initiated, whereby participants were instructed to match their movements with the ‘other participant’ (i.e., they were asked to perform in-phase coordination with the avatar). All other aspects were identical to the spontaneous coordination trial. During both trials, participants’ arm movements were captured (50 Hz) via tracking the hand-held controllers.

**Figure 1:**
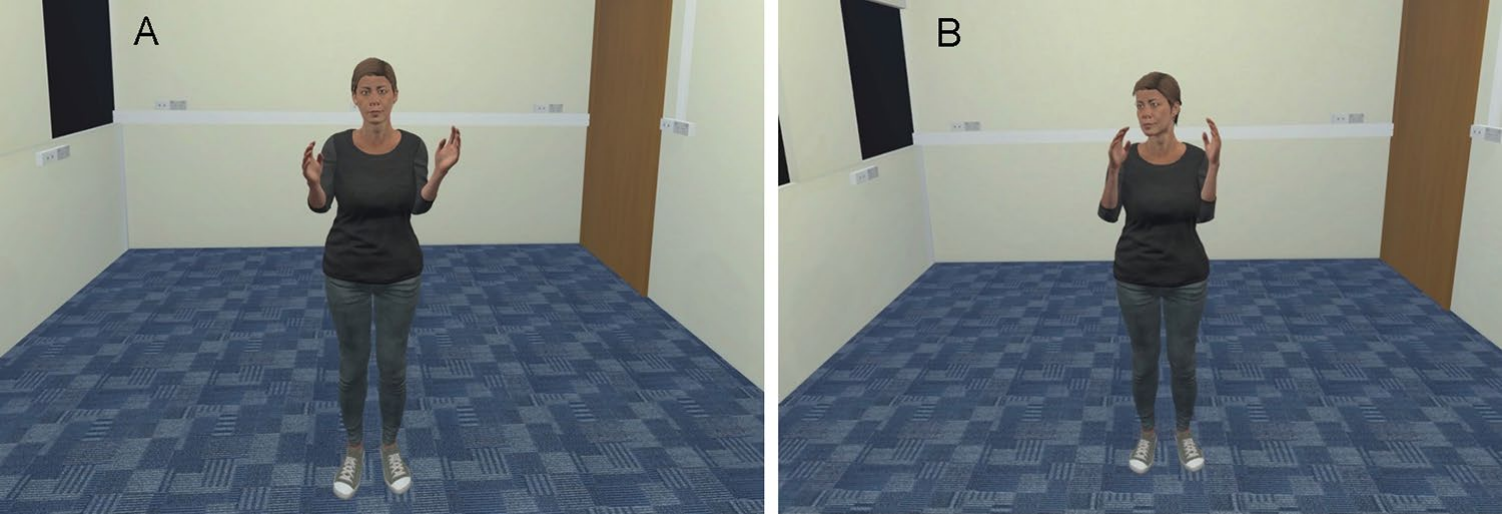
Participant view of the avatar during the interactive trials. (**A**) depicts the direct gaze condition; (**B**) depicts the averted gaze condition.

Importantly, each participant was randomly assigned to one of the two avatar gaze conditions, whereby the avatar either looked directly at the participant (i.e., the direct-gaze condition; Fig. 1, Panel A) or looked away and shifted their gaze frequently (i.e., the averted-gaze condition; Fig. 1, Panel B) for the duration of each trial. The avatar was created using Adobe Fuse CC (v 2017.1.0b) and rigged using Mixamo (www.mixamo.com) to resemble a typical university student in Australia (i.e., female, aged approximately 20–25 years, 1.64 m tall, casual clothing). To ensure realistic avatar behaviour, initially the movements of one of the experimenters were captured (100 Hz) using a Rokoko Smartsuit Pro and Rokoko Studio (Rokoko, Copenhagen, Denmark). The movements comprised arm curls performed in time with a metronome (84 bpm) and head movements such that it appeared as though the avatar averted their gaze away from the participant for the duration of each trial. Two sets of movements were captured and used to animate the avatar for each of the coordination trials, with the order of the pre-recorded movements counterbalanced across participants. For the direct gaze condition, the pre-recorded head movements were overridden using the Animator Controller in Unity3D, such that the avatar looked directly at the participant’s head and eye region for the duration of each trial.

As a manipulation check of differences in the social outcomes of the avatar gaze conditions, immediately after the intentional coordination trial participants completed a composite affiliation scale consisting of the Inclusion of Other in the Self Scale (IOS^45^), and seven items (e.g., “How much do you like the other participant?”, “How similar to you is the other participant?”) adapted from previous interpersonal coordination research^46^. Comparison between conditions revealed that, as expected, participants reported higher affiliation ratings in the direct (cf. averted) gaze condition, *t* (131) = 2.07, *p* = 0.04, *d* = 0.36.

Finally, participants were funnel debriefed to ascertain whether they believed the cover story (i.e., that the avatar was controlled by another participant). They were asked what they were trying to do while forming an impression of their partner, followed by what they thought the study was trying to achieve and whether anything seemed unusual. Participants who indicated that they did not believe another participant was present were excluded (*n* = 11). Finally, participants were debriefed as to the true purpose of the experiment and dismissed.

### Data reduction and estimation of coordination

Consistent with previous research^4,23^, the first 6 s of each trial were removed to eliminate any transient activity that may have occurred when initiating the arm curls. We then standardised the remainder of the trial to a length of 84 s. Each time series was centred around 0 and low-pass filtered using a 10 Hz Butterworth filter. Estimates of coordination between the movements of the right arm of the participant and the left arm of the avatar were calculated using custom Matlab scripts (github.com/xkiwilabs). Consideration of the opposite configuration (participant left arm, confederate right arm) revealed identical patterns of results. and therefore are not reported here.

Following relevant literature^25^ we focused on quantifying coordination across a range of complementary linear and non-linear metrics. Specifically, we used coordination stability (i.e., rho) as a global index of the degree to which participant and avatar arm movements were coordinated. We also considered indices that approximate key control parameters governing coordination^10,11^, namely frequency matching (i.e., coherence), coupling strength (i.e., MaxLine), and stochastic noise (i.e., %REC). In-depth treatment of these metrics is beyond the scope of this article (for further detail see^25,33,47,48^).

To estimate the global level of coordination, the distribution of relative phase relationships (*ϕ*) between participant and avatar arm movements was calculated using a Hilbert transform and normalised to a range of 0°–180°. The circular variance (rho) of the distribution of *ϕ* was calculated for each trial separately and standardised using a Fisher transformation. Rho provides an index of coordination stability ranging from 0 (i.e., no synchronisation) to 1 (i.e., perfect synchronisation).

To provide insight into the frequency matching parameter, average cross-spectral coherence was calculated for each trial. This measure is derived from a cross-spectral analysis of each time series whereby the obtained component frequencies are then correlated in the frequency domain (via calculation of a weighted average across the range of these frequencies). Coherence provides an index of frequency matching ranging from 0 (i.e., no coordination of movement frequency) to 1 (i.e., perfect coordination of movement frequency).

Finally, to estimate the non-linear structure of coordination, each timeseries was subject to a cross-recurrence quantification analysis (CRQA). Parameter settings for the delay and embedding dimensions were estimated using standard protocols^47^. A delay value was selected that corresponded to the first minimum of the average mutual information. The number of embedding dimensions was selected using the first minimum in a false nearest neighbour analysis. This resulted in an embedding dimension of 7 and a delay of 24. To maintain an average recurrence rate of approximately 5%, the radius was set to 15. The minimum line length was set to 2. Consistent with previous literature^25,48^, we then selected MaxLine and %REC as relevant coordination indices. These measures offer insight into any influence of SAD/ASD on coupling strength (MaxLine) or stochastic noise (%REC)^11^. For both measures, higher values correspond to more stable coordination dynamics.

### Linear mixed effects analyses

To address the research questions, linear mixed-effects models (LMMs) were constructed using the lme4 package^49^ and the lmerTest package^50^ in R (v 3.6.1). All predictor variables were centred prior to their inclusion in the models. For each model, coding for factorial variables is as follows: coordination [0 = spontaneous, 1 = intentional], avatar gaze [0 = direct, 1 = averted]. Degrees of freedom and* p*-values were estimated using Satterthwaite approximations. The random effects structure for each model comprised a by-participant random intercept. Interaction effects were decomposed by estimating Tukey-corrected post-hoc comparisons using the emmeans package^51^. The full dataset and analysis code is available on the Open Science Framework (https://osf.io/bez8f/).

## Results

### Partner gaze and interpersonal coordination

We first constructed a set of LMMs that separately considered the influence of avatar gaze (direct/averted) and coordination type (spontaneous/intentional) on each coordination measure (i.e., rho, coherence, %REC, MaxLine). Full results are presented in Supplementary Table 1.

**Figure 2:**
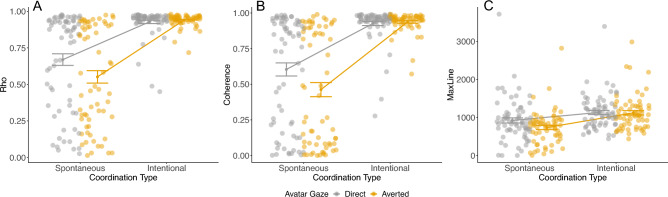
(**A**) Coordination stability (rho); (**B**) frequency matching (coherence); and (**C**) coupling (MaxLine), as a function of coordination type (spontaneous/intentional) and avatar gaze (direct/averted). For each graph, the error bars represent ± 1 SE.

For coordination stability (i.e., rho) and frequency matching (i.e., coherence), the models revealed main effects of avatar gaze (direct > averted) and coordination (spontaneous < intentional), which were qualified by interactions between these factors. Post hoc comparisons revealed higher levels of coordination stability and frequency matching when the avatar looked directly at the participant during spontaneous (rho: *b* = 0.12, SE = 0.04, *t* = 2.79, *p* = 0.03; coherence: *b* = 0.14, SE = 0.05, *t* = 2.88, *p* = 0.02), but not intentional (rho: *b* = − 0.01, SE = 0.04, *t* = − 0.27, *p* = 0.99; coherence: *b* = − 0.01, SE = 0.05, *t* = − 0.21, *p* = 0.99; Fig. 2, panels A and B), coordination.

For the cross-recurrence metrics, both models (i.e., %REC, MaxLine) revealed main effects of coordination (spontaneous < intentional). The MaxLine model also revealed a main effect of avatar gaze (direct > averted) and an interaction between these factors that approached significance. Figure 2 panel C suggests that, in line with the findings for rho and coherence, there was greater coupling strength in the direct (cf. averted) gaze condition for spontaneous coordination (*b* = 185.10, SE = 83.40, *t* = 2.22, *p* = 0.12). No comparable difference was observed when coordination was intentional (*b* = 10.70, SE = 83.70, *t* = 0.13, *p* = 0.99).

### Partner gaze, symptoms of psychopathology, and interpersonal coordination

Next, a set of LMMs considering the relationship between the questionnaire measures (i.e., LSAS/AQ) and each outcome of coordination (i.e., rho, coherence, %REC, MaxLine), were constructed. Each model specified fixed effects for one of the questionnaire measures (i.e., LSAS/AQ) alongside avatar gaze (direct/averted) and coordination type (spontaneous/intentional). Full results are presented in Supplementary Table 2.

**Figure 3:**
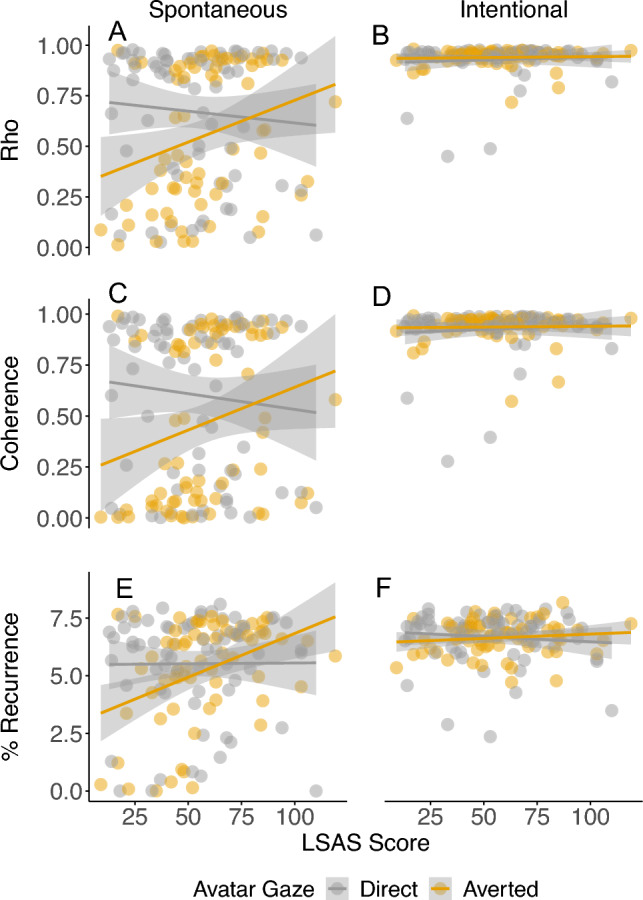
(Top row) The relationship between LSAS score and coordination stability (rho) as a function of avatar gaze (direct/averted) for (**A**) spontaneous and (**B**) intentional coordination; (Middle row) The relationship between LSAS score and frequency matching (coherence) as a function of avatar gaze (direct/averted) for (**C**) spontaneous and (**D**) intentional coordination; (Bottom row) The relationship between LSAS score and recurrent activity (%REC) as a function of avatar gaze (direct/averted) for (**E**) spontaneous and (**F**) intentional coordination. For each graph, the shaded area around each regression line represents ± 1 SE.

When considering LSAS and coordination stability (i.e., rho), the model revealed a two-way interaction between avatar gaze (direct/averted) and LSAS score, and a three-way interaction between avatar gaze, coordination type (spontaneous/intentional) and LSAS score. Post hoc comparisons revealed contrasting relationships between LSAS and rho as a function of avatar gaze in the spontaneous coordination condition (Fig. 3, panel A). Specifically, LSAS scores were positively related to rho when the avatar averted their gaze (*b* = 4.1 × 10^−3^, SE = 1.3 × 10^−3^, *t* = 3.13, *p* = 0.002), but not when gaze was direct (*b* = − 1.2 × 10^−3^, SE = 1.2 × 10^−3^, *t* = − 0.96, *p* = 0.34). No relationships were revealed in the intentional coordination condition (Fig. 3, panel B).

Equivalent patterns of effects were also revealed when considering the relationships between LSAS and coherence (direct gaze: *b* = − 1.5 × 10^−3^, SE = 1.4 × 10^−3^, *t* = − 1.09, *p* = 0.28; averted gaze: *b* = 4.2 × 10^−3^, SE = 1.6 × 10^−3^, *t* = 2.72, *p* = 0.007; Fig. 3, panels C and D), or LSAS and %REC (direct gaze: *b* = 6.9 × 10^−4^, SE = 0.01, *t* = 0.08, *p* = 0.94; averted gaze: *b* = 0.04, SE = 0.01, *t* = 4.10, *p* < 0.001; Fig. 3, panels E and F). No relationships were observed when considering MaxLine.

For AQ, no relationships were observed. We note however, that the signs of the coefficients (i.e., direction of the effects), were highly consistent between LSAS and AQ.

### Participant gaze

To gain insight into participant attentional patterns, we quantified the proportion of each trial in which the participant looked at the avatar. To identify systematic variation in participant gaze patterns we constructed a LMM with fixed effects for avatar gaze (direct/averted) and coordination type (spontaneous/intentional). The results revealed main effects of avatar gaze (direct > averted) and coordination (spontaneous < intentional), but no interaction between these factors. Full results are presented in Supplementary Table 3.

**Figure 4:**
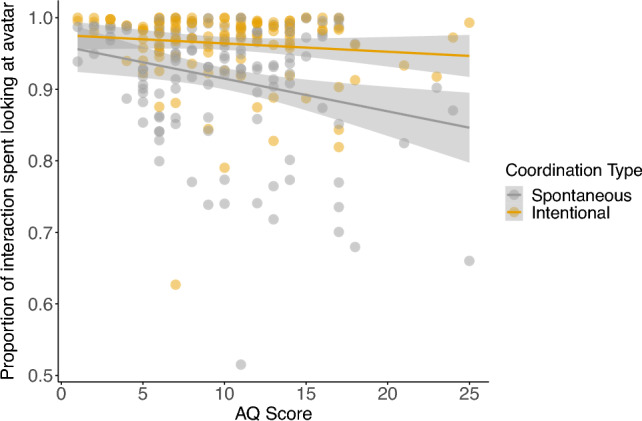
The relationship between proportion of the interaction spent looking at the avatar and AQ score as a function of coordination type (spontaneous/intentional). The shaded area around each regression line represents ± 1 SE.

We then constructed two LMMs to consider the relationship between participant gaze patterns and the questionnaire measures (i.e., LSAS/AQ). These models specified fixed effects for one of the questionnaire measures (i.e., LSAS/AQ) alongside avatar gaze (direct/averted) and coordination type (spontaneous/intentional). For LSAS, no relationships were observed. For AQ, however, the model revealed a main effect of AQ score, as well as an interaction between AQ and coordination type (Fig. 4). Post hoc comparisons revealed a negative relationship between AQ score and time spent looking at the avatar during spontaneous (*b* = − 3.8 × 10^−3^, SE = 1.3 × 10^−3^, *t* = − 2.88, *p* = 0.004), but not intentional coordination (*b* = − 9.0 × 10^−4^, SE = 1.3 × 10^−3^, *t* = − 0.68, *p* = 0.50).

### Participant gaze and interpersonal coordination

We then constructed a set of LMMs that considered the relationship between participant gaze and each outcome of coordination (i.e., rho, coherence, %REC, MaxLine). Each model specified fixed effects for participant gaze alongside avatar gaze (direct/averted) and coordination type (spontaneous/intentional). Full results are presented in Supplementary Table 4.

For coordination stability (i.e., rho) and frequency matching (i.e., coherence), the models revealed an effect of participant gaze, with higher levels of stability and frequency matching observed in participants who spent more time looking at the avatar.

For %REC, no relationships were observed, however the MaxLine model revealed an effect of participant gaze that approached significance. Higher levels of coupling observed for participants who spent more time looking at the avatar.

### Participant gaze, symptoms of psychopathology, and interpersonal coordination

Next, to explore any influence of symptoms of SAD/ASD on the relationship between participant gaze and coordination, LMMs were again constructed for each coordination measure (i.e., rho, coherence, %REC, MaxLine). Each model specified fixed effects for participant gaze and one of the questionnaire measures (i.e., LSAS/AQ) alongside avatar gaze (direct/averted) and coordination type (spontaneous/intentional). Full results are presented in Supplementary Table 5.

**Figure 5:**
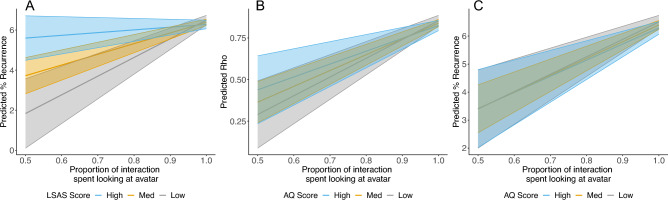
Relationships between the proportion of the interaction participants spent looking at the avatar and: (**A**) recurrent activity (%REC) as a function of LSAS score; (**B**) coordination stability (rho) as a function of AQ score; (**C**) recurrent activity as a function of AQ score. For each graph, the shaded area around each regression line represents ± 1 SE.

For LSAS, the %REC model revealed an interaction between participant gaze and LSAS score, with post hoc comparisons indicating a significant positive relationship between participant gaze and %REC at low (*b* = 9.46, SE = 3.79, *t* = 2.50, *p* = 0.01) and medium (*b* = 5.46, SE = 1.98, *t* = 2.75, *p* = 0.006) levels of LSAS, but not at high levels of LSAS (*b* = 1.46, SE = 2.38, *t* = 0.61, *p* = 0.54; Fig. 5 panel A). No relationships were observed for coordination stability (i.e., rho), frequency matching (i.e., coherence) or coupling (i.e., MaxLine).

For AQ, equivalent patterns of results were revealed when considering rho and %REC. Specifically, post hoc tests indicated a significant positive relationship between participant gaze and rho at low (*b* = 1.13, SE = 0.44, *t* = 2.58, *p* = 0.01) and medium (*b* = 0.95, SE = 0.27, *t* = 3.54, *p* < 0.001) levels of AQ, while this relationship approached significance at high levels of AQ (*b* = 0.77, SE = 0.44, *t* = 1.78, *p* = 0.08; Fig. 5, panel B). Similarly, post hoc tests indicated a significant positive relationship between participant gaze and %REC at low (*b* = 6.22, SE = 3.02, *t* = 2.06, *p* = 0.04) and medium (*b* = 5.99, SE = 1.86, *t* = 3.22, *p* = 0.001) levels of AQ, while this relationship also approached significance at high levels of AQ (*b* = 5.76, SE = 2.97, *t* = 1.94, *p* = 0.05; Fig. 5, panel C). No relationships were observed for frequency matching (i.e., coherence) or coupling (i.e., MaxLine).

## Discussion

This study investigated the influence of the gaze patterns of an interaction partner on the emergence of interpersonal coordination. In VR, participants performed a simple movement task with an avatar programmed to look either towards (i.e., direct gaze) or away (i.e., averted gaze) from them. We quantified the extent to which participants coordinated their movements with those of the avatar and assessed the impact of symptoms of SAD and ASD on key dynamical properties that characterise interpersonal coordination. A number of novel effects were revealed.

### Partner gaze and interpersonal coordination

Participants spontaneously coordinated with the avatar to a greater extent in the direct, compared to averted, gaze condition. To our knowledge, this is the first empirical demonstration that the gaze behaviour of an interaction partner can shape the spontaneous emergence of interpersonal coordination. This effect was robust across several measures of coordination. Averted partner gaze undermined overall coordination stability, an effect that was also revealed in disruptions to frequency matching and coupling strength. We suspect that differences in social context implied by the avatar’s attentional patterns (i.e., affiliative vs. non-affiliative) underlie this effect. Specifically, direct gaze may enhance coordination by inviting approach behaviour and opportunities for interaction, while averted gaze may undermine coordination by indicating disinterest and encouraging avoidance^13,15,16^. Indeed, our manipulation check revealed more positive impressions of the avatar in the direct gaze condition, lending support to this interpretation. More broadly, this finding adds a novel line of evidence to the literature indicating that interpersonal and contextual factors can serve to undermine, or enhance, coordinative actions^3,23,37^.

### Partner gaze, symptoms of psychopathology, and interpersonal coordination

We also found evidence that the relationship between symptoms of SAD and interpersonal coordination was impacted by partner gaze. In the spontaneous coordination condition, there was a *positive* association between coordination and SAD when the avatar averted their gaze, but no equivalent effect in the direct gaze condition. This relationship was again consistent across several measures of coordination (i.e., stability, frequency matching, recurrent activity). The direction of this effect was surprising and contrary to our predictions. Previous work has consistently reported disruptions to coordination as a function of social anxiety^4,24,25^. However, one exception stands out whereby^31^ social anxiety and behavioural coordination were negatively related during ‘closeness-generating’ conversations, but *positively* related during ‘small-talk’ conversations^29^. Together, this work suggests that factors that decrease perceived interpersonal scrutiny (i.e., a less intimate conversation or an averted gaze) may buffer against the typically deleterious effects of social anxiety on interpersonal coordination. No equivalent relationship was revealed between ASD and coordination.

Contemporary models of social behaviour may offer further insight into the positive association between symptoms of SAD and coordination. Contexts that threaten core social goals (e.g., belonging) can boost affiliative and ingratiating behaviour^21^, including interpersonal coordination^23^, as a means to (re)connect with an interaction partner. Although individuals with social anxiety tend to withdraw when feeling excluded^52^, they do show pro-social behaviours if ignored by meaningful others (e.g., potential future affiliates such as in-group members^53^). In the current study, participants anticipated a face-to-face meeting with their interaction partner (i.e., the ‘other participant’ allegedly controlling the avatar). As a result, the increased coordination associated with higher levels of symptoms of SAD may have served to reduce feelings of awkwardness or social distance associated with the upcoming interaction.

Notwithstanding this account, prominent models of social anxiety offer a complementary attentional explanation. Specifically, the vigilance-avoidance model^9^, suggests that averted (cf. direct) gaze patterns may allow more looking time for socially anxious individuals. People experiencing social anxiety are highly attentive to threating social cues such as direct gaze (i.e., vigilance), but rapidly orient away if eye-contact is made (i.e., avoidance). If eye contact never occurs due to a partner’s attention being directed elsewhere, individuals higher in social anxiety may maintain a vigilance strategy^54^ and continue to attend to their interaction partner. Future work should explore whether such patterns of sustained attention were related to the unexpected increase in coordination observed here.

### Participant gaze and interpersonal coordination

In terms of participant gaze, we found that more time spent looking at the avatar was equated with higher levels of coordination (i.e., increased stability, frequency-matching, and coupling strength). Although previous empirical work has manipulated attentional coupling and documented concomitant changes in coordination^6^, to our knowledge this is the first study to quantify this relationship via spontaneous attentional patterns during a social encounter. Consistent with prominent theories of social coordination^5^, the extent to which participants attended to the avatar (without prompting or instruction), impacted the degree to which coordination emerged.

The effect of participant gaze was consistent across all conditions despite both the experimental manipulations (i.e., coordination type and avatar gaze) influencing the time participants spent looking at the avatar. The additional attention participants paid to the avatar when the target of direct gaze, or when instructed to coordinate, did not further bolster coordination. While this may reflect the simplicity of the current task (i.e., coordinating arm curls may not demand a constant visual coupling to maintain adequate stability), contextual factors may also play a part. It has been observed that an intermittent visual coupling may characterise behaviour in social contexts, facilitating a balance between interdependence and individuality (i.e., a metastability of social behaviour^55^). We speculate that the current findings underscore this claim, suggesting that the attentional coupling necessary to sustain interpersonal coordination does not follow a simple ‘more-is-better’ relationship. Understanding how task constraints and context impact the link between attentional coupling and interpersonal coordination should be a priority within the relevant literature.

### Participant gaze, symptoms of psychopathology, and interpersonal coordination

The relationship between participant gaze and coordination also appeared to be constrained by symptoms of SAD and ASD. Higher levels of coordination (i.e., increased recurrent activity; %REC) were associated with more time spent looking at the avatar for individuals with low and medium levels of symptoms of SAD, but not for those with high levels. The same pattern of effects was observed for ASD, which also extended to estimates of coordination stability (i.e., rho). These results are important as they provide the initial empirical evidence that attentional patterns are directly implicated in the relationship between mental health and interpersonal coordination.

It will be beneficial to replicate and extend this work with more fine-grained measures of participant gaze behaviour. The areas of interest employed here (i.e., avatar vs. room) were designed to provide a preliminary examination of the association between naturalistic gaze patterns and coordination and did not permit consideration of precisely *where* the participant looked during the interaction. Although we observed consistent effects of participant gaze for symptoms of SAD and ASD, defining more precise areas of interest will allow potential distinctions in attentional behaviour associated with these disorders (e.g., vigilance/avoidance vs. gaze aversion^9,17^) to be captured. This may be important—fleeting eye contact (i.e., gaze vigilance) in contrast to no eye contact at all (i.e., gaze aversion) could denote a subtle difference in attentional coupling and concomitant variation to coordination as an interaction unfolds. Understanding the temporal contingencies that constrain the relationships between psychopathology, gaze, and coordination in social contexts is a promising avenue for future research.

### Theoretical, methodological, and practical implications

One factor that may limit the extent of the current results concerns the simplicity of the movement task we employed. Tasks with stable dynamics (e.g., arm curls) are straightforward to perform and resistant to perturbation^11^. On this basis, it has been suggested that the capacity for mental health symptoms to impact coordination increases as the stability of the task dynamics decrease^24^. Indeed, recent work demonstrates that system stability can act as a boundary condition on the relationships between symptoms of psychopathology and interpersonal coordination^25^. Of relevance to the current work, the associations uncovered between coordination, gaze, and mental health-related symptomology were observed in the spontaneous coordination condition only. Spontaneous (cf. intentional) coordination is dynamically less stable^6^. As a result, the task dynamics in the intentional coordination condition may not have provided the degree of instability necessary to capture perturbations driven by SAD or ASD symptomology. Future work should consider implementing tasks with lower levels of inherent stability (e.g., coordinating movements in orthogonal planes^56^).

As demonstrated here, employing VR enables precise manipulation of interpersonal behaviour while maintaining rigid experimental control. Although the generalisability of VR research warrants consideration^32^, the current study indicates strong consistencies with social behaviour in real-world contexts. Notably, participants found the interaction credible (only 7% suspected the avatar wasn’t human-controlled) and reported appropriate social outcomes (greater affiliation following direct gaze). Moreover, the dynamics of interpersonal coordination showed very strong consistency between virtual and real-world contexts. Nevertheless, while there is considerable evidence that VR is a valid tool for studying interpersonal coordination^31–34^, replication of the current findings in other contexts will serve to strengthen work in the area.

The current findings also point to avenues for targeting interventions to improve interpersonal coordination for those with symptoms of SAD. In therapeutic contexts, higher levels of coordination between therapists and clients are associated with better treatment outcomes^57^, while lower levels predict premature termination of therapy^58^. The current findings indicate that the relationship between social anxiety and coordination is malleable as a function of interaction partner behaviour. When the ‘intensity’ of the interpersonal exchange varied, so too does the nature of the relationship between coordination and SAD. Future work should focus on how clinicians can cultivate a therapeutic environment that encourages the emergence of interpersonal coordination.

## Conclusion

Interpersonal coordination provides a key foundation for successful interaction. Here we demonstrated that the averted gaze of an interaction partner disrupts the emergence of coordination (c.f. direct gaze). Of note, when partner gaze was averted, contrary to expectations we found a positive association between symptoms of social anxiety and coordination. These findings add weight to the growing body of evidence indicating that emergent patterns of coordination fluctuate as a function of meaningful changes in social context.

### Supplementary Information

Supplementary Information.

## Data Availability

The full dataset and analysis code is available on the Open Science Framework (https://osf.io/bez8f/).
