# Supplementary material for: Partner gaze shapes the relationship between symptoms of psychopathology and interpersonal coordination
Source: Sci Rep. 2024 Jun 21;14:14288. doi: 10.1038/s41598-024-65139-5 (PMC11192736; doi:10.1038/s41598-024-65139-5)
Supplement: Supplementary file 1 — Supplementary Information. [file 41598_2024_65139_MOESM1_ESM.docx]

SUPPLEMENTARY MATERIALS

**Partner gaze shapes the relationship between symptoms of psychopathology and interpersonal coordination**

M. C. Macpherson,^1^ A. J. Brown,^1^ R. W. Kallen,^2^ M. J. Richardson,^2^ & L. K. Miles^1^

^1^ School of Psychological Science, University of Western Australia, Australia

^2^ School of Psychological Sciences and Centre for Elite Performance, Expertise and Training, Macquarie University, Australia

APPENDIX A

Full Results for Each Model

*Partner gaze and interpersonal coordination*

| **Table S1** | | | | | |  |
| --- | --- | --- | --- | --- | --- | --- |
| Fixed effects of coordination type (spontaneous/intentional) and avatar gaze (direct/averted) on each index of coordination (i.e., Rho, Coherence, %REC, MaxLine). | | | | | | |
| **DV** | **Predictors** | **B** | **SE** | **df** | ***t*** | ***p*** |
| Rho | (Intercept) | 0.670 | 0.029 | 258.403 | 22.800 | <.001 |
|  | Coordination | 0.258 | 0.040 | 131.627 | 6.530 | <.001 |
|  | Avatar gaze | -0.118 | 0.042 | 258.403 | -2.789 | .006 |
|  | Coord*avatar gaze | 0.130 | 0.057 | 131.138 | 2.280 | .024 |
|  |  |  |  |  |  |  |
| Coherence | (Intercept) | 0.604 | 0.034 | 257.913 | 17.689 | <.001 |
|  | Coordination | 0.322 | 0.046 | 131.567 | 7.043 | <.001 |
|  | Avatar gaze | -0.142 | 0.049 | 257.913 | -2.876 | .004 |
|  | Coord*avatar gaze | 0.152 | 0.066 | 131.078 | 2.312 | .022 |
|  |  |  |  |  |  |  |
| %REC | (Intercept) | 5.517 | 0.208 | 237.705 | 26.515 | <.001 |
|  | Coordination | 1.170 | 0.245 | 130.886 | 4.774 | <.001 |
|  | Avatar gaze | -0.303 | 0.300 | 237.705 | -1.011 | .313 |
|  | Coord*avatar gaze | 0.260 | 0.352 | 130.442 | 0.738 | .462 |
|  |  |  |  |  |  |  |
| MaxLine | (Intercept) | 921.348 | 57.864 | 238.106 | 15.923 | <.001 |
|  | Coordination | 210.634 | 68.224 | 131.322 | 3.087 | .002 |
|  | Avatar gaze | -185.082 | 83.414 | 238.106 | -2.219 | .027 |
|  | Coord*avatar gaze | 174.366 | 98.106 | 130.877 | 1.777 | .078 |

*Partner gaze, symptoms of psychopathology, and interpersonal coordination*

| **Table S2** | | | | | | | | | | | |  | |  | |
| --- | --- | --- | --- | --- | --- | --- | --- | --- | --- | --- | --- | --- | --- | --- | --- |
| Fixed effects of the relationship between coordination type (spontaneous/intentional), avatar gaze (direct/averted) and questionnaire measure (LSAS/AQ score), on each index of coordination (i.e., Rho, Coherence, %REC, MaxLine). | | | | | | | | | | | | | | | |
|  | LSAS | | | | | |  | AQ | | | | | | | |
| **DV** | **Predictors** | **B** | **SE** | **df** | ***t*** | ***p*** |  | **Predictors** | **B** | **SE** | **df** | | ***t*** | | ***p*** |
| Rho | (Intercept) | 0.668 | 0.029 | 254.221 | 22.950 | <.001 |  | (Intercept) | 0.665 | 0.030 | 254.276 | | 22.030 | | <.001 |
|  | Coordination | 0.261 | 0.039 | 129.458 | 6.679 | <.001 |  | Coordination | 0.264 | 0.040 | 129.061 | | 6.515 | | <.001 |
|  | Avatar gaze | -0.124 | 0.042 | 254.221 | -2.961 | .003 |  | Avatar gaze | -0.123 | 0.044 | 254.276 | | -2.827 | | .005 |
|  | LSAS | -0.001 | 0.001 | 254.221 | -0.960 | .338 |  | AQ | -0.005 | 0.006 | 254.276 | | -0.790 | | .430 |
|  | Coord*avatar gaze | 0.135 | 0.056 | 129.041 | 2.405 | .018 |  | Coord*avatar gaze | 0.136 | 0.058 | 128.844 | | 2.329 | | .021 |
|  | Coord*LSAS | 0.001 | 0.002 | 129.495 | 0.896 | .372 |  | Coord*AQ | 0.005 | 0.008 | 130.721 | | 0.612 | | .542 |
|  | Avatar gaze*LSAS | 0.005 | 0.002 | 254.221 | 2.955 | .003 |  | Avatar gaze*AQ | 0.014 | 0.009 | 254.276 | | 1.554 | | .121 |
|  | Coord*avatar gaze*LSAS | -0.006 | 0.002 | 129.038 | -2.287 | .024 |  | Coord*avatar gaze*AQ | -0.016 | 0.012 | 129.638 | | -1.273 | | .205 |
|  |  |  |  |  |  |  |  |  |  |  |  | |  | |  |
| Coherence | (Intercept) | 0.601 | 0.034 | 253.669 | 17.708 | <.001 |  | (Intercept) | 0.597 | 0.035 | 253.692 | | 17.036 | | <.001 |
|  | Coordination | 0.326 | 0.045 | 129.367 | 7.184 | <.001 |  | Coordination | 0.329 | 0.047 | 128.958 | | 7.050 | | <.001 |
|  | Avatar gaze | -0.147 | 0.049 | 253.669 | -3.005 | .003 |  | Avatar gaze | -0.147 | 0.051 | 253.692 | | -2.900 | | .004 |
|  | LSAS | -0.002 | 0.001 | 253.669 | -1.091 | .276 |  | AQ | -0.007 | 0.007 | 253.692 | | -0.925 | | .356 |
|  | Coord*avatar gaze | 0.157 | 0.065 | 128.951 | 2.405 | .018 |  | Coord*avatar gaze | 0.160 | 0.067 | 128.742 | | 2.368 | | .019 |
|  | Coord*LSAS | 0.002 | 0.002 | 129.404 | 1.034 | .303 |  | Coord*AQ | 0.007 | 0.010 | 130.614 | | 0.714 | | .477 |
|  | Avatar gaze*LSAS | 0.006 | 0.002 | 253.669 | 2.740 | .007 |  | Avatar gaze*AQ | 0.018 | 0.011 | 253.692 | | 1.671 | | .096 |
|  | Coord*avatar gaze*LSAS | -0.006 | 0.003 | 128.948 | -2.174 | .032 |  | Coord*avatar gaze*AQ | -0.020 | 0.014 | 129.534 | | -1.424 | | .157 |
|  |  |  |  |  |  |  |  |  |  |  |  | |  | |  |
| %REC | (Intercept) | 5.518 | 0.204 | 232.812 | 27.104 | <.001 |  | (Intercept) | 5.517 | 0.214 | 233.767 | | 25.810 | | <.001 |
|  | Coordination | 1.161 | 0.238 | 128.707 | 4.880 | <.001 |  | Coordination | 1.144 | 0.251 | 128.391 | | 4.564 | | <.001 |
|  | Avatar gaze | -0.380 | 0.294 | 232.812 | -1.295 | .197 |  | Avatar gaze | -0.380 | 0.309 | 233.767 | | -1.229 | | .220 |
|  | LSAS | 0.001 | 0.008 | 232.812 | 0.081 | .936 |  | AQ | 0.000 | 0.044 | 233.767 | | -0.005 | | .996 |
|  | Coord*avatar gaze | 0.337 | 0.342 | 128.331 | 0.983 | .327 |  | Coord*avatar gaze | 0.354 | 0.362 | 128.194 | | 0.980 | | .329 |
|  | Coord*LSAS | -0.006 | 0.010 | 128.740 | -0.563 | .574 |  | Coord*AQ | -0.028 | 0.052 | 129.893 | | -0.525 | | .601 |
|  | Avatar gaze*LSAS | 0.037 | 0.013 | 232.812 | 2.967 | .003 |  | Avatar gaze*AQ | 0.068 | 0.064 | 233.767 | | 1.062 | | .289 |
|  | Coord*avatar gaze*LSAS | -0.029 | 0.015 | 128.328 | -1.954 | .053 |  | Coord*avatar gaze*AQ | -0.033 | 0.076 | 128.915 | | -0.441 | | .660 |
|  |  |  |  |  |  |  |  |  |  |  |  | |  | |  |
| MaxLine | (Intercept) | 921.244 | 58.203 | 234.809 | 15.828 | <.001 |  | (Intercept) | 919.730 | 59.468 | 234.036 | | 15.466 | | <.001 |
|  | Coordination | 207.173 | 68.712 | 129.198 | 3.015 | .003 |  | Coordination | 197.386 | 69.768 | 128.845 | | 2.829 | | .005 |
|  | Avatar gaze | -190.499 | 83.940 | 234.809 | -2.269 | .024 |  | Avatar gaze | -185.619 | 85.921 | 234.036 | | -2.160 | | .032 |
|  | LSAS | -0.056 | 2.428 | 234.809 | -0.023 | .982 |  | AQ | -1.551 | 12.331 | 234.036 | | -0.126 | | .900 |
|  | Coord*avatar gaze | 179.494 | 98.883 | 128.818 | 1.815 | .072 |  | Coord*avatar gaze | 201.668 | 100.689 | 128.649 | | 2.003 | | .047 |
|  | Coord*LSAS | -2.284 | 2.867 | 129.231 | -0.797 | .427 |  | Coord*AQ | -14.209 | 14.591 | 130.349 | | -0.974 | | .332 |
|  | Avatar gaze*LSAS | 2.826 | 3.594 | 234.809 | 0.786 | .433 |  | Avatar gaze*AQ | 3.467 | 17.871 | 234.036 | | 0.194 | | .846 |
|  | Coord*avatar gaze*LSAS | 1.448 | 4.234 | 128.815 | 0.342 | .733 |  | Coord*avatar gaze*AQ | 1.710 | 21.029 | 129.370 | | 0.081 | | .935 |

*Participant gaze*

| **Table S3** | | | | | |
| --- | --- | --- | --- | --- | --- |
| Fixed effects of the relationship between coordination type (spontaneous/intentional), avatar gaze (direct/averted) and questionnaire measure (LSAS/AQ score), on time spent looking at the avatar. | | | | | |
| **Predictors** | **B** | **SE** | **df** | ***t*** | ***p*** |
| (Intercept) | 0.931 | 0.009 | 228.822 | 109.531 | <.001 |
| Coordination | 0.040 | 0.010 | 124.429 | 3.969 | <.001 |
| Avatar gaze | -0.036 | 0.013 | 229.439 | -2.845 | .005 |
| Coord*avatar gaze | 0.020 | 0.015 | 124.949 | 1.362 | .176 |
|  |  |  |  |  |  |
| (Intercept) | 0.930 | 0.009 | 225.234 | 108.626 | <.001 |
| Coordination | 0.041 | 0.010 | 122.407 | 4.026 | <.001 |
| Avatar gaze | -0.034 | 0.013 | 225.776 | -2.724 | .007 |
| LSAS | -0.001 | 0.000 | 225.234 | -1.406 | .161 |
| Coord*avatar gaze | 0.020 | 0.015 | 122.866 | 1.339 | .183 |
| Coord*LSAS | 0.000 | 0.000 | 122.407 | 0.936 | .351 |
| Avatar gaze*LSAS | 0.000 | 0.001 | 225.347 | 0.845 | .399 |
| Coord*avatar gaze*LSAS | -0.001 | 0.001 | 122.502 | -1.033 | .304 |
|  |  |  |  |  |  |
| (Intercept) | 0.926 | 0.009 | 224.785 | 107.937 | <.001 |
| Coordination | 0.045 | 0.010 | 122.384 | 4.454 | <.001 |
| Avatar gaze | -0.028 | 0.013 | 225.210 | -2.180 | .030 |
| AQ | -0.005 | 0.002 | 224.785 | -2.951 | .004 |
| Coord*avatar gaze | 0.014 | 0.015 | 122.735 | 0.955 | .341 |
| Coord*AQ | 0.005 | 0.002 | 122.384 | 2.436 | .016 |
| Avatar gaze*AQ | 0.003 | 0.003 | 225.006 | 1.132 | .259 |
| Coord*avatar gaze*AQ | -0.005 | 0.003 | 122.566 | -1.450 | .150 |

*Participant gaze and interpersonal coordination*

| **Table S4** | | | | | | |
| --- | --- | --- | --- | --- | --- | --- |
| Fixed effects of coordination type (spontaneous/intentional), avatar gaze (direct/averted) and participant gaze, on each index of coordination (i.e., Rho, Coherence, %REC, MaxLine). | | | | | | |
| **DV** | **Predictors** | **B** | **SE** | **df** | ***t*** | ***p*** |
| Rho | (Intercept) | 0.677 | 0.028 | 241.199 | 23.923 | <.001 |
|  | Coordination | 0.221 | 0.044 | 158.039 | 5.067 | <.001 |
|  | Avatar gaze | -0.045 | 0.045 | 242.803 | -1.011 | .313 |
|  | Ppt gaze | 0.812 | 0.326 | 242.366 | 2.488 | .014 |
|  | Coord*avatar gaze | 0.084 | 0.062 | 153.251 | 1.353 | .178 |
|  | Coord*ppt gaze | 0.121 | 0.773 | 216.640 | 0.156 | .876 |
|  | Avatar gaze*ppt gaze | 0.571 | 0.488 | 242.516 | 1.170 | .243 |
|  | Coord*avatar gaze*ppt gaze | -1.393 | 0.979 | 211.553 | -1.423 | .156 |
|  |  |  |  |  |  |  |
| Coherence | (Intercept) | 0.613 | 0.033 | 240.465 | 18.399 | <.001 |
|  | Coordination | 0.276 | 0.051 | 157.401 | 5.390 | <.001 |
|  | Avatar gaze | -0.067 | 0.052 | 242.371 | -1.277 | .203 |
|  | Ppt gaze | 0.893 | 0.384 | 241.850 | 2.329 | .021 |
|  | Coord*avatar gaze | 0.112 | 0.073 | 152.663 | 1.538 | .126 |
|  | Coord*ppt gaze | 0.292 | 0.906 | 215.091 | 0.322 | .748 |
|  | Avatar gaze*ppt gaze | 0.685 | 0.573 | 242.028 | 1.194 | .234 |
|  | Coord*avatar gaze*ppt gaze | -1.691 | 1.148 | 209.920 | -1.473 | .142 |
|  |  |  |  |  |  |  |
| %REC | (Intercept) | 5.512 | 0.201 | 222.616 | 27.380 | <.001 |
|  | Coordination | 0.981 | 0.281 | 156.740 | 3.496 | .001 |
|  | Avatar gaze | 0.196 | 0.315 | 231.099 | 0.620 | .536 |
|  | Ppt gaze | 2.824 | 2.222 | 228.460 | 1.271 | .205 |
|  | Coord*avatar gaze | -0.043 | 0.397 | 153.157 | -0.109 | .914 |
|  | Coord*ppt gaze | 2.989 | 5.122 | 194.141 | 0.584 | .560 |
|  | Avatar gaze*ppt gaze | 3.552 | 3.325 | 229.273 | 1.068 | .286 |
|  | Coord*avatar gaze*ppt gaze | -7.438 | 6.465 | 188.967 | -1.150 | .251 |
|  |  |  |  |  |  |  |
| MaxLine | (Intercept) | 928.150 | 57.871 | 225.297 | 16.038 | <.001 |
|  | Coordination | 151.073 | 81.778 | 156.589 | 1.847 | .067 |
|  | Avatar gaze | -107.462 | 90.728 | 232.899 | -1.184 | .237 |
|  | Ppt gaze | 1241.514 | 643.662 | 230.584 | 1.929 | .055 |
|  | Coord*avatar gaze | 156.187 | 115.841 | 152.849 | 1.348 | .180 |
|  | Coord*ppt gaze | 387.408 | 1488.275 | 196.509 | 0.260 | .795 |
|  | Avatar gaze*ppt gaze | 183.386 | 962.785 | 231.309 | 0.190 | .849 |
|  | Coord*avatar gaze*ppt gaze | -447.115 | 1879.283 | 191.258 | -0.238 | .812 |

*Participant gaze, symptoms of psychopathology, and interpersonal coordination*

| **Table S5** | | | | | | | | | | | |  |  | |
| --- | --- | --- | --- | --- | --- | --- | --- | --- | --- | --- | --- | --- | --- | --- |
| Fixed effects of coordination type (spontaneous/intentional), avatar gaze (direct/averted), questionnaire measures (LSAS/AQ) and participant gaze, on coordination stability (i.e., Rho) and frequency matching (i.e., Coherence). | | | | | | | | | | | | | |  |
|  | LSAS | | | | | |  | AQ | | | | | |  |
| **DV** | **Predictors** | **B** | **SE** | **df** | ***t*** | ***p*** |  | **Predictors** | **B** | **SE** | **df** | ***t*** | ***p*** |  |
| Rho | (Intercept) | 0.673 | 0.029 | 233.582 | 23.579 | <.001 |  | (Intercept) | 0.661 | 0.030 | 234.218 | 21.963 | <.001 |  |
|  | Coordination | 0.222 | 0.044 | 152.658 | 5.020 | <.001 |  | Coordination | 0.235 | 0.046 | 152.407 | 5.161 | <.001 |  |
|  | Avatar gaze | -0.043 | 0.045 | 234.969 | -0.965 | .336 |  | Avatar gaze | -0.032 | 0.046 | 235.040 | -0.701 | .484 |  |
|  | LSAS | -0.001 | 0.001 | 233.559 | -0.703 | .483 |  | AQ | -0.006 | 0.007 | 235.488 | -0.868 | .386 |  |
|  | Ppt gaze | 0.915 | 0.387 | 234.900 | 2.362 | .019 |  | Ppt gaze | 1.093 | 0.371 | 235.002 | 2.946 | .004 |  |
|  | Coord*avatar gaze | 0.087 | 0.064 | 155.422 | 1.354 | .178 |  | Coord*avatar gaze | 0.076 | 0.064 | 146.454 | 1.173 | .243 |  |
|  | Coord*LSAS | 0.002 | 0.002 | 159.146 | 0.918 | .360 |  | Coord*AQ | 0.005 | 0.013 | 200.313 | 0.382 | .703 |  |
|  | Avatar gaze*LSAS | 0.002 | 0.002 | 235.090 | 1.059 | .291 |  | Avatar gaze*AQ | 0.011 | 0.010 | 235.427 | 1.116 | .266 |  |
|  | Coord*ppt gaze | 0.111 | 0.808 | 201.224 | 0.137 | .891 |  | Coord*ppt gaze | -0.092 | 0.804 | 201.390 | -0.115 | .909 |  |
|  | Avatar gaze*ppt gaze | 0.609 | 0.534 | 234.813 | 1.140 | .256 |  | Avatar gaze*ppt gaze | 0.518 | 0.569 | 234.327 | 0.911 | .363 |  |
|  | LSAS*ppt gaze | -0.011 | 0.017 | 234.936 | -0.666 | .506 |  | AQ*ppt gaze | -0.114 | 0.055 | 234.091 | -2.071 | .039 |  |
|  | Coord*avatar gaze*LSAS | -0.003 | 0.003 | 166.357 | -1.022 | .308 |  | Coord*avatar gaze*AQ | -0.012 | 0.016 | 179.131 | -0.729 | .467 |  |
|  | Coord*avatar gaze*ppt gaze | -1.597 | 1.093 | 201.305 | -1.461 | .146 |  | Coord*avatar gaze*ppt gaze | -1.432 | 1.034 | 202.997 | -1.385 | .168 |  |
|  | Coord*LSAS*ppt gaze | -0.006 | 0.039 | 204.498 | -0.158 | .874 |  | Coord*AQ*ppt gaze | 0.146 | 0.239 | 225.952 | 0.613 | .540 |  |
|  | Avatar gaze*LSAS*ppt gaze | -0.029 | 0.026 | 234.309 | -1.102 | .272 |  | Avatar gaze*AQ*ppt gaze | 0.043 | 0.109 | 235.022 | 0.398 | .691 |  |
|  | Coord*avatar gaze*LSAS*ppt gaze | 0.051 | 0.060 | 210.418 | 0.860 | .391 |  | Coord*avatar gaze*AQ*ppt gaze | -0.070 | 0.279 | 216.541 | -0.252 | .801 |  |
|  |  |  |  |  |  |  |  |  |  |  |  |  |  |  |
| Coherence | (Intercept) | 0.607 | 0.034 | 232.908 | 18.018 | <.001 |  | (Intercept) | 0.598 | 0.036 | 233.680 | 16.807 | <.001 |  |
|  | Coordination | 0.278 | 0.052 | 152.309 | 5.352 | <.001 |  | Coordination | 0.289 | 0.054 | 152.487 | 5.388 | <.001 |  |
|  | Avatar gaze | -0.064 | 0.053 | 234.563 | -1.200 | .231 |  | Avatar gaze | -0.056 | 0.055 | 234.658 | -1.032 | .303 |  |
|  | LSAS | -0.001 | 0.001 | 232.881 | -0.879 | .381 |  | AQ | -0.007 | 0.008 | 235.191 | -0.848 | .398 |  |
|  | Ppt gaze | 1.035 | 0.456 | 234.477 | 2.268 | .024 |  | Ppt gaze | 1.085 | 0.438 | 234.610 | 2.477 | .014 |  |
|  | Coord*avatar gaze | 0.113 | 0.076 | 155.130 | 1.491 | .138 |  | Coord*avatar gaze | 0.107 | 0.076 | 146.608 | 1.419 | .158 |  |
|  | Coord*LSAS | 0.002 | 0.002 | 158.762 | 1.028 | .305 |  | Coord*AQ | 0.005 | 0.015 | 199.738 | 0.352 | .725 |  |
|  | Avatar gaze*LSAS | 0.003 | 0.002 | 234.707 | 1.091 | .276 |  | Avatar gaze*AQ | 0.014 | 0.012 | 235.118 | 1.170 | .243 |  |
|  | Coord*ppt gaze | 0.256 | 0.951 | 199.720 | 0.270 | .788 |  | Coord*ppt gaze | 0.171 | 0.947 | 200.122 | 0.181 | .857 |  |
|  | Avatar gaze*ppt gaze | 0.687 | 0.630 | 234.374 | 1.091 | .277 |  | Avatar gaze*ppt gaze | 0.757 | 0.671 | 233.809 | 1.129 | .260 |  |
|  | LSAS*ppt gaze | -0.016 | 0.020 | 234.521 | -0.794 | .428 |  | AQ*ppt gaze | -0.091 | 0.065 | 233.528 | -1.399 | .163 |  |
|  | Coord*avatar gaze*LSAS | -0.004 | 0.003 | 165.976 | -1.027 | .306 |  | Coord*avatar gaze*AQ | -0.015 | 0.019 | 178.932 | -0.801 | .424 |  |
|  | Coord*avatar gaze*ppt gaze | -1.853 | 1.286 | 199.791 | -1.441 | .151 |  | Coord*avatar gaze*ppt gaze | -1.872 | 1.217 | 201.694 | -1.538 | .126 |  |
|  | Coord*LSAS*ppt gaze | -0.002 | 0.045 | 203.057 | -0.048 | .962 |  | Coord*AQ*ppt gaze | 0.135 | 0.281 | 224.847 | 0.481 | .631 |  |
|  | Avatar gaze*LSAS*ppt gaze | -0.025 | 0.031 | 233.772 | -0.817 | .415 |  | Avatar gaze*AQ*ppt gaze | 0.012 | 0.129 | 234.635 | 0.097 | .923 |  |
|  | Coord*avatar gaze*LSAS*ppt gaze | 0.046 | 0.070 | 208.966 | 0.654 | .514 |  | Coord*avatar gaze*AQ*ppt gaze | -0.039 | 0.329 | 215.303 | -0.118 | .907 |  |

| **Table S6** | | | | | | | | | | | |  |  | |
| --- | --- | --- | --- | --- | --- | --- | --- | --- | --- | --- | --- | --- | --- | --- |
| Fixed effects of coordination type (spontaneous/intentional), avatar gaze (direct/averted), questionnaire measures (LSAS/AQ) and participant gaze, on each recurrence metric (i.e., %REC, MaxLine). | | | | | | | | | | | | | |  |
|  | LSAS | | | | | |  | AQ | | | | | |  |
| **DV** | **Predictors** | **B** | **SE** | **df** | ***t*** | ***p*** |  | **Predictors** | **B** | **SE** | **df** | ***t*** | ***p*** | |
| %REC | (Intercept) | 5.472 | 0.198 | 216.403 | 27.590 | <.001 |  | (Intercept) | 5.404 | 0.211 | 218.434 | 25.566 | <.001 | |
|  | Coordination | 0.956 | 0.278 | 149.871 | 3.438 | .001 |  | Coordination | 0.986 | 0.287 | 149.530 | 3.436 | .001 | |
|  | Avatar gaze | 0.196 | 0.310 | 223.937 | 0.631 | .529 |  | Avatar gaze | 0.291 | 0.324 | 223.282 | 0.896 | .371 | |
|  | LSAS | 0.003 | 0.008 | 216.284 | 0.339 | .735 |  | AQ | -0.019 | 0.047 | 225.930 | -0.402 | .688 | |
|  | Ppt gaze | 6.593 | 2.584 | 223.212 | 2.552 | .011 |  | Ppt gaze | 6.354 | 2.497 | 222.729 | 2.545 | .012 | |
|  | Coord*avatar gaze | 0.062 | 0.405 | 153.215 | 0.153 | .878 |  | Coord*avatar gaze | -0.032 | 0.404 | 144.849 | -0.080 | .936 | |
|  | Coord*LSAS | 0.001 | 0.012 | 155.471 | 0.053 | .957 |  | Coord*AQ | -0.063 | 0.083 | 185.840 | -0.767 | .444 | |
|  | Avatar gaze*LSAS | 0.015 | 0.013 | 224.556 | 1.114 | .266 |  | Avatar gaze*AQ | 0.044 | 0.070 | 225.784 | 0.628 | .531 | |
|  | Coord*ppt gaze | 0.534 | 5.213 | 178.796 | 0.102 | .918 |  | Coord*ppt gaze | 1.689 | 5.212 | 178.004 | 0.324 | .746 | |
|  | Avatar gaze*ppt gaze | 0.652 | 3.565 | 222.722 | 0.183 | .855 |  | Avatar gaze*ppt gaze | 1.483 | 3.814 | 218.816 | 0.389 | .698 | |
|  | LSAS*ppt gaze | -0.291 | 0.111 | 223.415 | -2.624 | .009 |  | AQ*ppt gaze | -1.106 | 0.369 | 217.522 | -2.995 | .003 | |
|  | Coord*avatar gaze*LSAS | -0.020 | 0.019 | 161.977 | -1.051 | .295 |  | Coord*avatar gaze*AQ | 0.038 | 0.102 | 170.945 | 0.378 | .706 | |
|  | Coord*avatar gaze*ppt gaze | -7.403 | 7.049 | 178.708 | -1.050 | .295 |  | Coord*avatar gaze*ppt gaze | -7.813 | 6.706 | 178.955 | -1.165 | .246 | |
|  | Coord*LSAS*ppt gaze | 0.053 | 0.250 | 182.573 | 0.213 | .831 |  | Coord*AQ*ppt gaze | 2.556 | 1.578 | 201.254 | 1.620 | .107 | |
|  | Avatar gaze*LSAS*ppt gaze | 0.035 | 0.175 | 219.967 | 0.200 | .841 |  | Avatar gaze*AQ*ppt gaze | 0.618 | 0.734 | 223.032 | 0.842 | .400 | |
|  | Coord*avatar gaze*LSAS*ppt gaze | 0.308 | 0.387 | 187.199 | 0.795 | .427 |  | Coord*avatar gaze*AQ*ppt gaze | -2.115 | 1.829 | 191.642 | -1.157 | .249 | |
|  |  |  |  |  |  |  |  |  |  |  |  |  |  | |
| MaxLine | (Intercept) | 922.953 | 58.602 | 217.817 | 15.750 | <.001 |  | (Intercept) | 914.990 | 61.261 | 221.675 | 14.936 | <.001 | |
|  | Coordination | 150.664 | 82.917 | 149.249 | 1.817 | .071 |  | Coordination | 134.017 | 84.848 | 149.817 | 1.579 | .116 | |
|  | Avatar gaze | -94.887 | 91.649 | 224.909 | -1.035 | .302 |  | Avatar gaze | -82.952 | 94.026 | 225.791 | -0.882 | .379 | |
|  | LSAS | 0.657 | 2.438 | 217.705 | 0.270 | .788 |  | AQ | -0.023 | 13.620 | 228.041 | -0.002 | .999 | |
|  | Ppt gaze | 1828.095 | 766.934 | 224.263 | 2.384 | .018 |  | Ppt gaze | 1893.518 | 730.897 | 225.387 | 2.591 | .010 | |
|  | Coord*avatar gaze | 140.162 | 120.778 | 152.589 | 1.160 | .248 |  | Coord*avatar gaze | 178.829 | 119.522 | 144.916 | 1.496 | .137 | |
|  | Coord*LSAS | -2.647 | 3.595 | 154.955 | -0.736 | .463 |  | Coord*AQ | -29.043 | 24.325 | 188.269 | -1.194 | .234 | |
|  | Avatar gaze*LSAS | -2.968 | 3.984 | 225.496 | -0.745 | .457 |  | Avatar gaze*AQ | -9.255 | 20.252 | 227.872 | -0.457 | .648 | |
|  | Coord*ppt gaze | -181.201 | 1551.385 | 179.733 | -0.117 | .907 |  | Coord*ppt gaze | 145.126 | 1535.255 | 181.352 | 0.095 | .925 | |
|  | Avatar gaze*ppt gaze | -111.540 | 1058.149 | 223.804 | -0.105 | .916 |  | Avatar gaze*ppt gaze | 9.861 | 1117.113 | 222.051 | 0.009 | .993 | |
|  | LSAS*ppt gaze | -42.899 | 32.972 | 224.454 | -1.301 | .195 |  | AQ*ppt gaze | -172.678 | 108.171 | 220.935 | -1.596 | .112 | |
|  | Coord*avatar gaze*LSAS | 6.430 | 5.575 | 161.592 | 1.153 | .250 |  | Coord*avatar gaze*AQ | 25.206 | 29.955 | 172.263 | 0.841 | .401 | |
|  | Coord*avatar gaze*ppt gaze | 7.914 | 2097.876 | 179.656 | 0.004 | .997 |  | Coord*avatar gaze*ppt gaze | -838.762 | 1974.951 | 182.413 | -0.425 | .672 | |
|  | Coord*LSAS*ppt gaze | 38.469 | 74.392 | 183.533 | 0.517 | .606 |  | Coord*AQ*ppt gaze | 517.868 | 463.449 | 205.532 | 1.117 | .265 | |
|  | Avatar gaze*LSAS*ppt gaze | -42.003 | 51.885 | 221.211 | -0.810 | .419 |  | Avatar gaze*AQ*ppt gaze | -30.693 | 214.899 | 225.609 | -0.143 | .887 | |
|  | Coord*avatar gaze*LSAS*ppt gaze | 26.307 | 115.160 | 188.346 | 0.228 | .820 |  | Coord*avatar gaze*AQ*ppt gaze | -386.242 | 537.660 | 195.598 | -0.718 | .473 | |
